# Synergistic effects of cementitious nanocomposites grouts fused with epoxy coatings for restoring concrete structures

**DOI:** 10.1038/s41598-025-23802-5

**Published:** 2025-11-17

**Authors:** A. K. Roopa, Dhananjay Ambale, A. M. Hunashyal, Shantharam Patil

**Affiliations:** 1 School of Civil Engineering, KLE Technological University, Hubballi, Karnataka 580030 India; 2 Manipal School of Architecture and Planning, Manipal Academy of Higher Education, Manipal, 576104 Karnataka India

**Keywords:** Multiwall carbon nanotube, Nanocomposite grouting, Epoxy coating, Mechanical properties, Retrofitting, Engineering, Materials science

## Abstract

Grouting techniques are used in retrofitting structures to improve the structural integrity and performance of existing structures. The recent era of nanotechnology enabled the development of high-performance nanocomposite based cementitious grouts, epoxy grouts, and polyurethane grouts which enhance the strengths, durability, and life span on structures. However, use of only nanocomposite cement grouting is inadequate to bridge micro-cracks, densify the interfacial transition zone (ITZ) after retrofitting. In this study, a novel two-step hybrid crack restoring approach for concrete structures is introduced by combining injected Multi-Walled Carbon Nanotube (MWCNT) reinforced cementitious grout for nanoscale crack-bridging and ITZ densification with an epoxy coating for surface sealing for treating cracks as an integrated nano–micro–surface system. The main objective of the study is improving the mechanical performance and structural integrity of retrofitted concrete structures by assessing their synergistic effect on load-carrying capacity, deformation, and durability. The experimental study is carried out to evaluate the mechanical properties such as load carrying capacity, stress, strain, ductility, sulfate attack and influence of elevated temperature. The results demonstrate that the rupture strength of crack-restored specimens increased by 27.27, 33.33, and 20% compared to plain concrete specimens subjected to controlled room temperature, elevated temperature of 400 °C, and sulfate exposure, respectively. The microstructural analysis reveals that nanoparticle fill-up cracks in the cement composite, enhancing interaction at the interfacial zone. This study highlights the effectiveness of nanomaterial-infused grouting techniques as an innovative and efficient solution for structural retrofitting by offering improved strength, longevity, and resistance to environmental conditions.

## Introduction

The deterioration of concrete structures due to excessive loading, exposure to aggressive environmental conditions and aging over time has led to the need for advanced retrofitting techniques that enhance structural integrity and prolong service life^1,2^. Retrofitting techniques involve strengthening existing structures to restore their load-carrying capacity, improve durability, and mitigate future degradation^3,4^. Several retrofitting techniques are used to strengthen the existing structure, such as wrapping carbon fibre reinforced polymer, post tensioning cables to reinforce the structure, bracing supports to prevent collapse and epoxy injection to repair the cracks^5^. These methods are used to increase stability and avoid further damage of structures^6,7^. Grouting techniques facilitate the injection of cement-based grout material into the pores to restore the cracks of the concrete surface. Grouting materials should possess good bond strength, injectability, fluidity, durability, and impermeability^8,9^. Therefore, growing demand for sustainable and long-lasting infrastructure has intensified the need for advanced materials and techniques in civil engineering.

At the present, nanotechnology has emerged as a promising approach to enhance the mechanical strength and durability of retrofitted concrete structures^10,11^. Nanomaterials, such as multi-walled carbon nanotubes (MWCNTs), nano-silica, titanium dioxide (TiO_2_) and nano-alumina, are incorporated into concrete to improve mechanical strength, durability, and resistance to environmental degradation^12,13^. Studies have shown that incorporating nanomaterials into cementitious composites improves compressive strength, flexural strength, and fracture toughness by refining the microstructure and improving the interfacial bonding between the cement matrix and aggregates^14,15^. Additionally, nanomaterials act as nucleation sites for the formation of calcium silicate hydrate (C-S–H) gel which is responsible for the strength and durability of cementitious materials^16^. Previous studies show that MWCNTs and Nano silica are used as crack filling materials to improve compressive strength, tensile strength and load carrying of concrete structures. Similarly, the ultimate load carrying capacity of concrete specimen was increased up to 105% by the addition of 0.25% carbon fibre and 121.65% by the addition of 0.75% MWCNT into concrete mix respectively^17^. The percentage of CNT in the matrix has a significant impact on the interfacial interaction between the CNT and the cement matrix which improves the mechanical and electrical properties^18^. The flexural strength of concrete is increased by 12.9, 15.8, and 22.0% with the addition of 0.1, 0.3, and 0.5% of MWCNT, while resistivity decreased by 16.9, 19.7, and 40.3% respectively^19^. Multi-wall carbon nanotubes and carbon fibres were well dispersed in the cement matrix which enhanced the mechanical and electrical properties^20,21^. However, some studies suggest that incorporation of MWCNT into matrix improves mechanical properties up to a critical concentration, beyond which agglomeration occurs, negatively affecting strength and durability^22^. The higher concentration of CNT leads to the clustering that weakness the cement matrix and reduces the mechanical properties. This is mainly due to the high surface area and strong Van der Waals forces between nanotubes decrease the workability leading to lower strength^23,24^. Hence the optimum percentage of nano materials plays a crucial role in ensuring the required strength and workability.

In addition to the above, the nano materials are used to prepare the high-performance grouting material for retrofitting applications. Recent studies suggest that incorporating multi-walled carbon nanotubes (MWCNTs) into cementitious grouts enhances their mechanical and electrical properties and significantly improves the interfacial bond between the grout and concrete^25,26^. Additionally, MWCNT-epoxy composites have demonstrated significant improvements in crack-bridging ability, corrosion resistance, and thermal stability, making them a promising solution for structural rehabilitation^27,28^. Also, the incorporation of nanomaterials enhances the self-healing capabilities of cementitious composites, allowing microcracks to be sealed through continued hydration reactions^29,30^. In addition to cementitious grouts, epoxy coatings play a crucial role in retrofitting applications by providing a protective barrier against environmental degradation, moisture ingress, and chemical attacks^31,32^. The MWCNT based epoxy coatings enhances the mechanical strength, adhesion, and crack resistance compared to traditional epoxy coating^33^. The incorporation of MWCNTs into epoxy matrices improves load transfer efficiency, reduces microcracking, and enhances impact resistance, making them highly suitable for structural repair applications^34^.

From the above literature, cement-based grout injection or polymer/epoxy sealing are commonly used to restore stiffness and impermeability in concrete crack structures. However, use of single nanocomposite-based grouting methods is ineffective to bridge micro-cracks, stabilize the interfacial transition zone, and maintain performance after repairs when structures are subjected high temperatures or sulfate-rich conditions. MWCNT modified cement composite improve mechanical strength through crack-bridging and percolation. Still, most studies focus on bulk composites at normal conditions rather than the durability of pre-cracked elements after repair. Despite significant progress in the field of nanotechnology in construction materials, there are still detailed investigations required on the integrated effect of MWCNT-based cementitious grouts fused with epoxy coatings for structural retrofitting applications. This study aims to provide an innovative and efficient method of grouting to extend the service life of damaged concrete structures using nanotechnology. Hence, the proposed work addresses the two steps hybrid approach for treating crack surface by injecting MWCNT reinforced cement grout to bridge and densify cracks at the nano to micro scale, along with a thin epoxy coating to reduce surface permeability and limit of ion ingress. The innovation focuses on concrete crack surfaces repaired as a coupled system rather than a bulk MWCNT composite or epoxy coating, where post repair durability is measured after exposure to high temperatures of 400 °C and sulfates using pre-cracked and controlled damaged samples. The primary objectives of the proposed study are to develop the cement nanocomposite grouting to repair the concrete and epoxy based nano coating to seal the concrete crack surface to enhance the performance of grouting by improving flexural strength, toughness and ductility properties at high temperature rather than at normal conditions. The experimental results show that, hybrid MWCNT cementitious grouting with epoxy coating approach proves to be effective method to maintain post-repair flexural strength at elevate temperature and sulfate conditions to enhance the resilience and longevity of rehabilitated concrete structures.

## Experimental investigation

### Material used

In this study Ordinary Portland Cement (OPC) 43 grade cement, natural river sand, and 12 mm coarse aggregation are used for casting samples. The multiwalled carbon nanotubes are used to prepare cement-based grouting to repair the cracks of concrete surface, while MWCNT based epoxy is used as coating material on to seal the retrofitted concrete surface as dual-purpose applications.

#### Concrete

OPC 43 grade cement is used for grouting and the preparation of concrete samples. The M25 grade concrete with a 1:1:2 proportion are used to cast the test specimen with a water to cement ratio of 0.45, zone III fine aggregate, and 10 mm downsize coarse aggregate confirming Indian Standard 10,262–2019 code. The concrete specimens are cured for 28 days before carrying out the experiential test.

#### Multi walled carbon nanotubes

The MWCNT were used in preparation of grouting paste due to their high aspect ratio and strong interfacial bonding with the surrounding matrix material. When incorporated MWCNTs disperse evenly, forming a network that reinforces the structure which enhances load transfer and improves mechanical properties. Additionally, MWCNTs high surface area facilitates better adhesion to the substrate, enhancing durability and conductivity. MWCNTs were procured from Tokyo Chemical Industry (India) Pvt. Ltd and properties are shown in Table 1.

**Table 1:** Physical characteristics of MWCNT.

Item	Properties
Purity	> 92%
Colour	Black
Inner diameter	5–12 nm
Outer diameter	10–25 nm
Length	15–35 um
Thermal conductivity	1600–3200 W/m°C
Density	2200 kg/m^3^

#### Epoxy resin

Epoxy resin is an effective synthetic polymer used for coating, laminating and binding purpose. It possesses durable and adhesive properties, when it is mixed with hardener. Hence, in the present study epoxy resin is used as coating material for cracked concrete surface to improve strength and resist abrasion. Table 2 represents the epoxy resins.

**Table 2:** Physical characteristics of epoxy resins.

Properties	Epoxy specification
Colour	Resin part: White, Hardener part: Gray Resin with Hardener: Light Gray
Mixing ratio (Resin: Hardener)	9:1
Density	1160 kg/m^3^
Viscosity	9500–12,000 MPa

## Methodology

### Casting of specimens

**Fig. 1:**
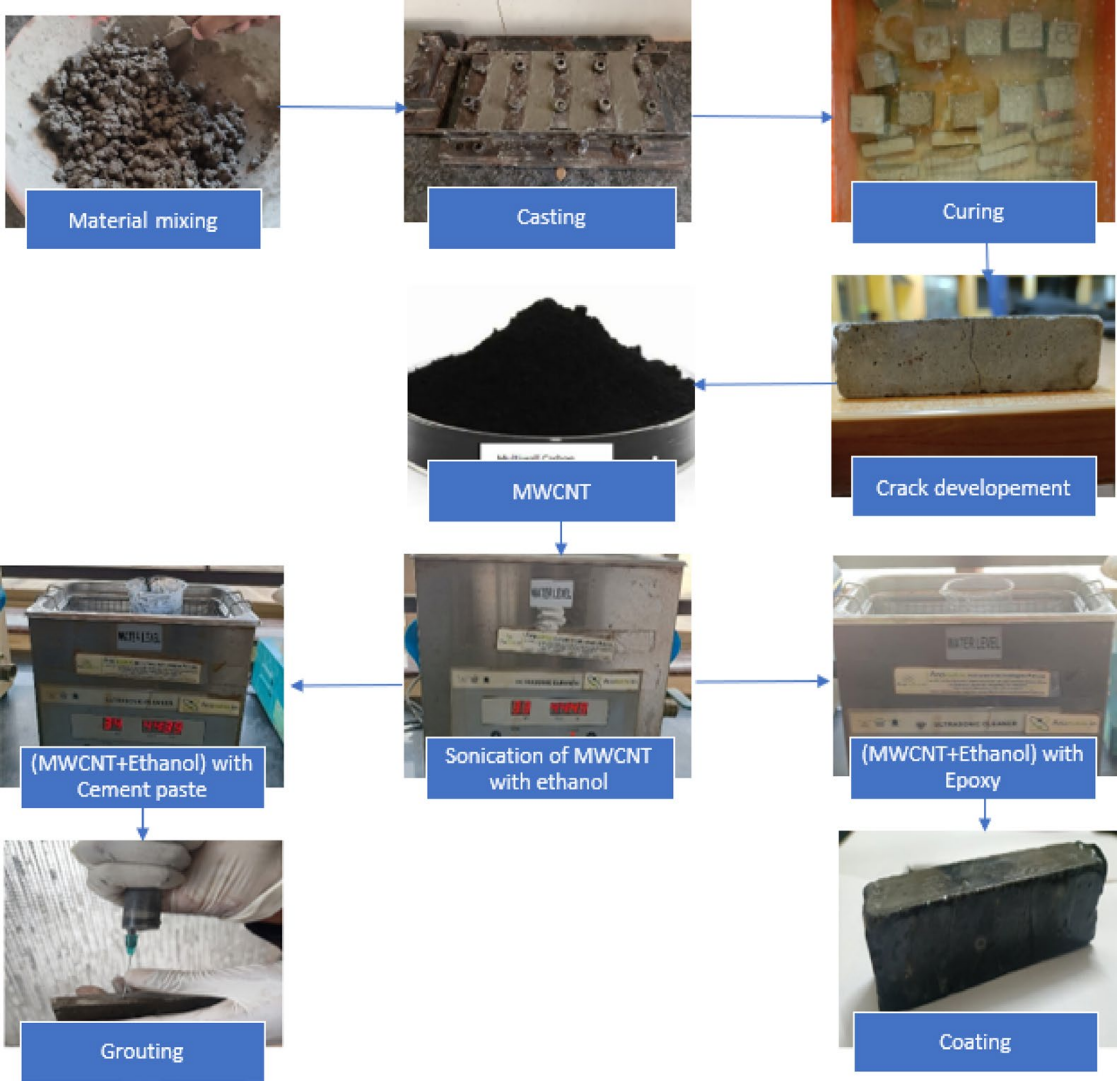
Process of sample preparation for grouting with coating.

The proposed study involves the development of novel cement based nanocomposite grouting to repair the cracked concrete surface fused with MWCNT epoxy coating. Therefore, experimental investigations include assessing the behaviour of the above grouting material subjected to flexural loading under elevated temperature and durability characteristics due to sulfate attack. According to literature studies, incorporating higher percentages of MWCNTs can lead to strength reduction due to agglomeration and poor dispersion, which negatively impact the microstructure and workability of cementitious materials^35,36^. Therefore, in this study, 0.1% MWCNT (by weight of cement) was used as threshold concentration to prepare the grouting paste. The grout slurry is prepared by adding 0.1% MWCNT by weight of cement into cement base followed by pre dispersion of MWCNT using ethanol and then sonication is carried out at high intensity with the help of ultra sonicator. The uniform dispersion of MWCNT is achieved by double sonication method as explained above to reduce the repulsive is Van der Waals force between nano particles. The 0.5% weigh of MWCNT sodium dodecyl sulfate used as surfactants by 0.5% weigh of MWCNT to maintain workability. A similar approach is adopted to prepare the epoxy based MWCNT coating by incorporating 1% MWCNT concentration of by weight of epoxy to seal the grouting materials on cracked surface. Table 3 presents the compositions of beam specimens cast with dimensions of 20 × 20 × 80 mm and cured for 28 days. Although ASTM C348 specifies 40 × 40 × 160 mm prisms, the present study adapted scaled-down geometry while maintaining the three-point bending procedure, span ratio, and modulus of rupture calculation as per ASTM C348 principles^37^. Reduced-size prisms were intentionally adapted for technical and practical reasons, such as improved MWCNT dispersion and homogeneity at smaller cross-sections, reduced material requirement given the high cost of nanomaterials, and alignment with prior studies using mini-prisms to assess cement nanocomposite performance^38,39^. Accordingly, the flexural results are reported as obtained from a procedure adapted from ASTM C348 using reduced specimen dimensions of cement nanocomposites. The loading carrying capacity of the beam grouted with various composition is evaluated by inducing cracks by applying 85% of the failure load of rupture in plain concrete beams. The cracks on beams surface are filled with cement based nanocomposite grouting and then sealed by epoxy coating. Figure 1 demonstrate the procedure of casting of sample for experiments. The retrofitting of cracked induced beam is carried out by grouting followed by coating approach. Initially, the grouting process begins with the removal of any residual moisture by removing water from the cracks and beams with air drying. Cracks were filled properly by injecting the MWCNT-cementitious grouting slurry into them at regular intervals. Later on, as a second treatment epoxy-MWCNT coating was applied to hinder the segregation of grouting material and to achieve strong adherence for concrete surface. These concrete specimen specimens were cured for seven days at room temperature epoxy coatings to ensure the good bonding of the hardened concrete before carrying out the experimental tests.

**Table 3:** Composition details of concrete beam specimens.

Specimen name	Composition details
PC	Plain Concrete
M-GC	Cracked Concrete beam MWCNT based coating fused with epoxy coating
PC-ET	Plain Concrete subjected to elevated temperature
M-GC-ET	Cracked Concrete beam MWCNT based coating fused with epoxy coating subjected to elevated temperature
PC-SA	Plain Concrete exposed to sulfate attack
M-GC-SA	Cracked Concrete beam MWCNT based coating fused with epoxy coating exposed to sulfate attack

### Experimental tests

The experimental tests such as flexural test, durability test and micro structural analysis were carried out to assess the efficacy of grouting and coating materials on concrete specimens along with effect of elevated temperature.

#### Flexural test

**Fig. 2:**
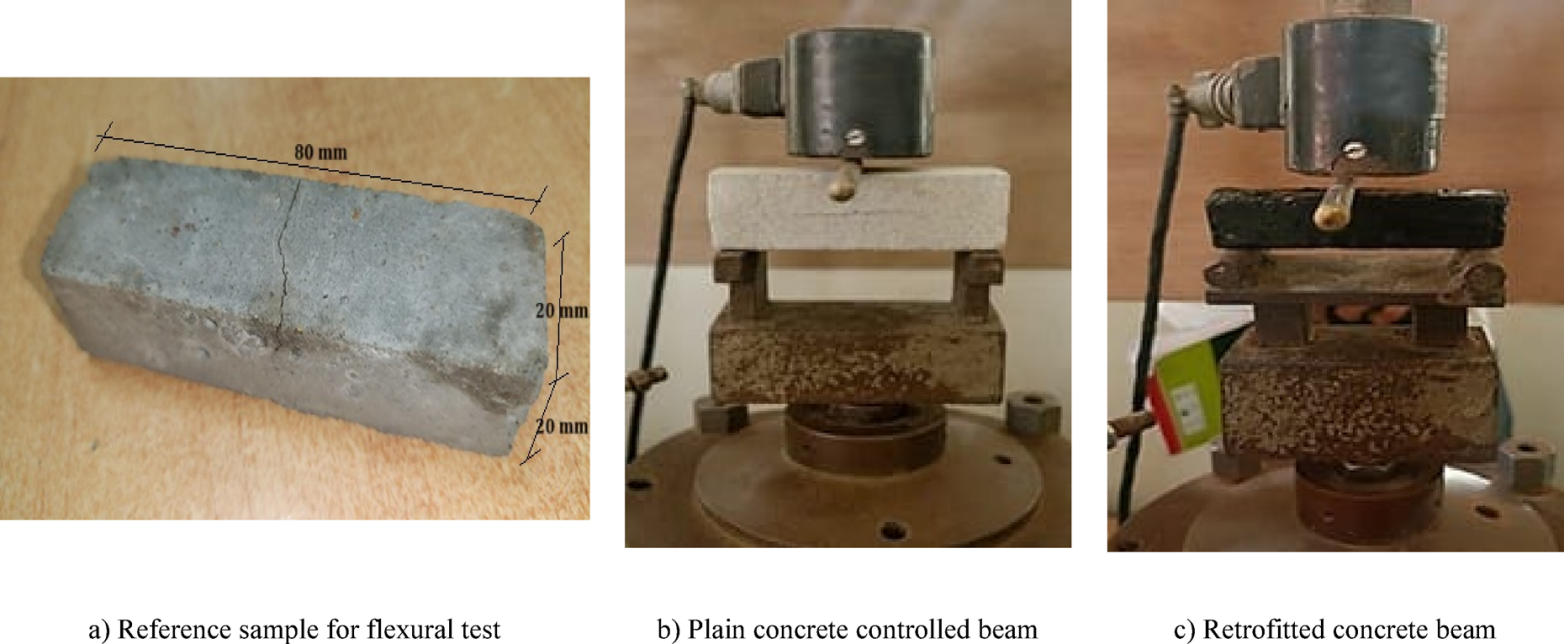
Flexural strength test set up.

The above-mentioned concrete specimens were subjected to a three-point flexural test to evaluate their mechanical properties as shown in Fig. 2. Each specimen was tested by applying 0.05 mm/min regulated strain rate using a load frame with a 10 kN capacity to perform the bending test. Shear forces were created inside the beam sections by the application of concentrated loads at the specimen’s centre, which caused a distribution of moments. From the experimental test results, stress stain graphs are plotted to the behaviors of sample under loading. The flexural strength of concrete specimen and modulus of rapture at failure load is determined by using Eq. (1) as below.

1 $$f_{b} = \frac{PL}{{bd^{2} }}$$

where P is the Ultimate load; L is the effective length; and d and b are the depth and width of the specimens, respectively.

Similarly, the bending stiffness (EI) of the beams was obtained from the load–deflection response using the Eq. (2).

2 $$f_{b} = \frac{{PL^{3} }}{48EI}$$

where, *P* is the applied load at mid-span, *L* is the span, and *Δ* is the corresponding deflection and EI is flexural rigidity. After obtaining flexural strength and bending stiffness from the experimental test results, the flexural toughness and ductility index are calculated as explained section "Flexural toughness" and "Ductility index" respectively.

#### Elevated temperature test

**Fig. 3:**
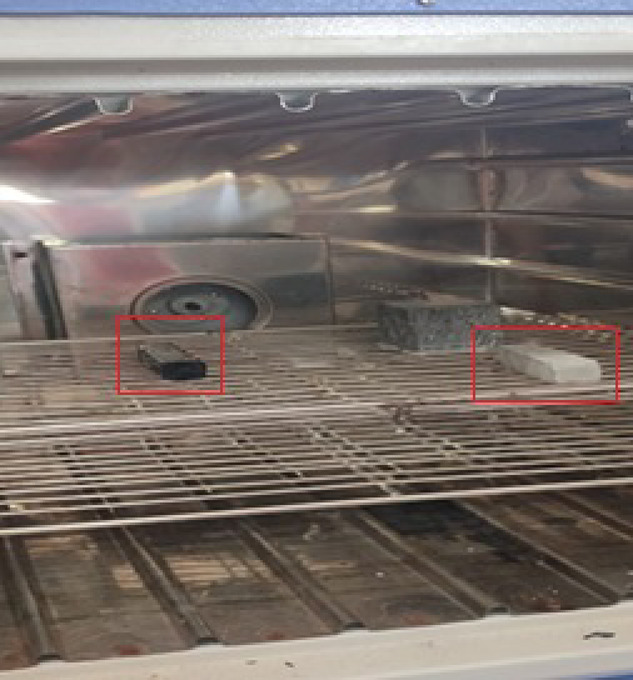
Concrete specimen subjected to 400 °C temperature in oven.

The elevated temperature test are conducted on various concrete specimens by keeping them into the oven at a controlled thermal exposure as shown in Fig. 3. Plain concrete beams (PC-ET) and crack-restored beams with MWCNT grouting and epoxy coating (M-GC-ET) were placed in a laboratory oven and heated to 400 °C for 24 h^40^. After heating, the specimens were gradually cooled to room temperature to prevent thermal shock. This thermal conditioning simulated elevated temperature conditions and allowed the assessment of thermal degradation effects on strength and durability. Later on, flexural test was conducted to evaluate the effect of elevated temperature on both PC-ET and M-GC-ET specimens to determine changes in load-carrying capacity, modulus of rupture, and ductility parameters.

#### Durability test

**Fig. 4:**
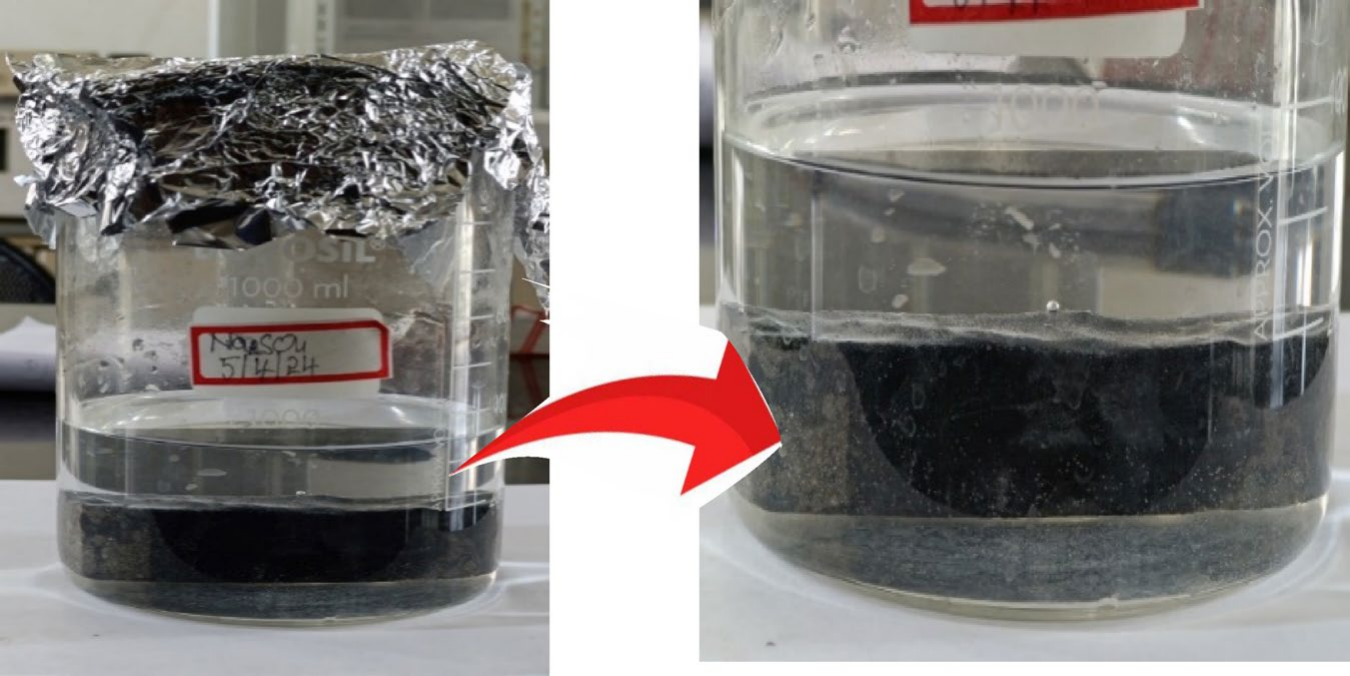
Specimens exposed to sulfate attack.

The durability test is carried out to know behavior retrofitted concrete specimen subjected to water permeability, sulfate attack under environmental conditions. As demonstrated in Fig. 4 concrete samples PC-SA and M-GC-SA were submerged in a 10% sodium sulfate solution for 15 days and recuperated for three days at room temperature. Lately, flexural tests have been carried on these samples to evaluate the effects of sulfate exposure and structural integrity. EDTA titration method was employed to determine the sulfate concentration in the solution. The procedure to determine the concentration of sulfate ions in the concrete using EDTA titration method starts with measuring 50 mL of the solution into a volume of 250 mL conical flask, followed by the small addition of HCL acid. Barium chloride (10–30 mL) was then added dropwise from a burette and shaking was applied to minimize precipitate adsorption. The volume (V_1_) added was noted. Next, the flask was rinsed with 1–2 mL of water and heated for 2 min then cooled. A solution comprising Mg-EDTA, hydroxylamine, ethyl alcohol, triethanolamine, hydrochloride, buffer and indicator was added while shaking the container. The EDTA standard solution was then titrated until the color from fuchsia changes to pure blue and the volume consumed (V_2_) was recorded. Lastly, 5 mL sample was pipetted into the flask which was followed by the addition of water and HCL acid for acidulation. Triethanolamine, hydroxylamine hydrochloride, buffer and indicator were added. Titration with EDTA was performed until color changes and the consumed volume (V_0_) was noted. This method helps for the determination of sulfate concentration in the solution^41,42^.

The Eq. (3) is used to calculate the of sulfate ion concentration in the analyzed solution^19^.

3 $$\rho_{{SO_{4}^{2 - } }} = \frac{{c_{1} V_{1} - c_{2} \left( {V_{2} - V_{0} } \right)}}{{V_{L0} }}$$

Here, c_1_-concentration of the barium chloride (BaCl_2_) standard solution, set at 0.05 mol/L. v_1_-volume of BaCl_2_ solution used during sample measurement. c_2_-concentration of the ethylenediaminetetraacetic acid (EDTA) standard solution, fixed at 0.02 mol/L. v_2_-volume of EDTA solution consumed during sample measurement, while v_0_-volume of EDTA solution consumed when determining the total concentration of calcium ions (Ca^2+^) and magnesium ions (Mg^2+^) in a 5 mL sample.

#### Microstructural analysis

The microstructural characterization is carried out on cured concrete specimen using scanning electron microscope (SEM) to assess the grouting and coating morphology and hybrid effects of nano particles in concrete samples. The Hitachi SU3500 Scanning Electron Microscope with resolution of 3.0 nm at 30 kV and a magnification range from 5 × to 300,000 × is used to study the concrete specimen. The morphology of specimen is recorded by the tungsten filament electron with pressure of 120 Pa.

## Results and discussions

From the above experimental study mechanical properties such as behaviour of lad-deformation, stress–strain characteristics, flexural strength, durability properties exposed to sulfate attack, effect of elevated temperature, toughness, ductility index, flexural stiffness and modulus of elasticity are studied as below.

### Flexural strength

**Fig. 5:**
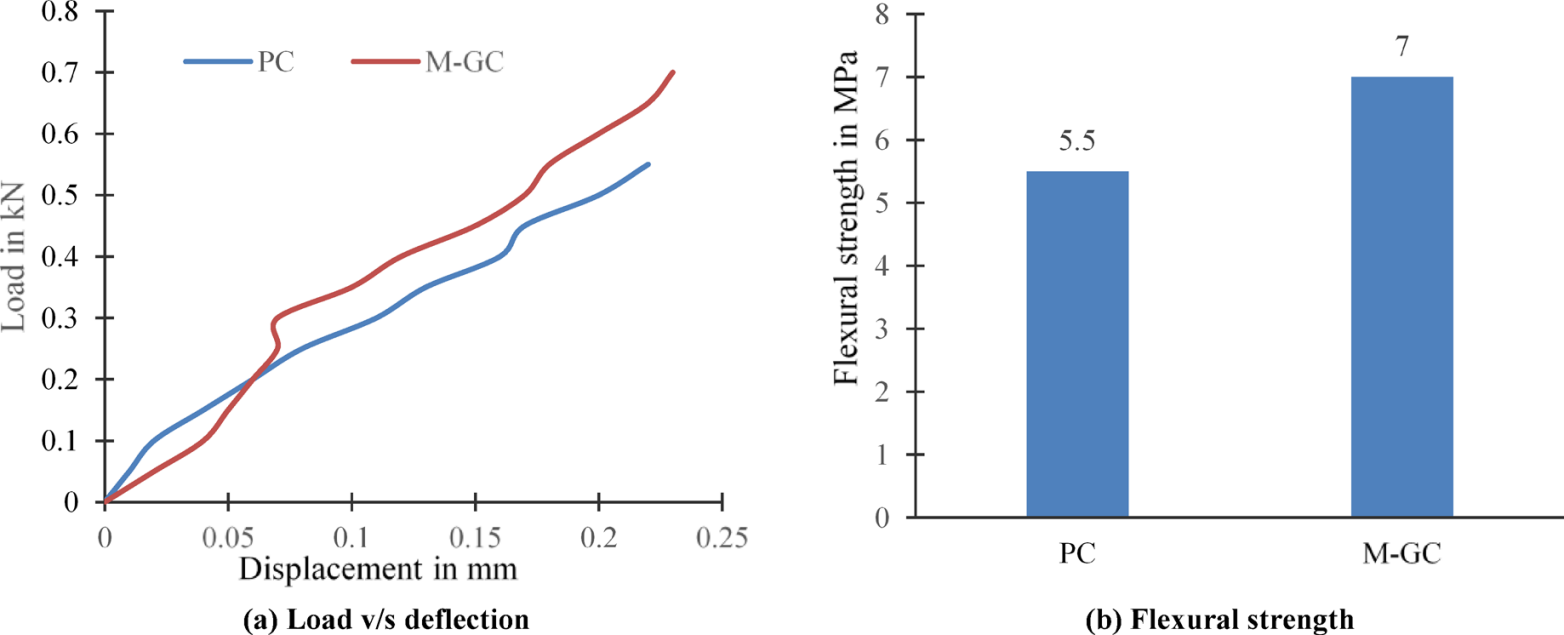
Specimen subjected to flexural loading.

From the experimental test, The load versus deflection graph is drawn as shown in Fig. 5a. The values shown represent the average of three specimens tested under identical conditions. This figure shows the lack of distinct yield points that demonstrate the material’s plastic behaviour, due to the addition of MWCNTs into the grouting and coating materials. These MWCNTs into the cement paste and epoxy enhanced the load-bearing capacity by bridging microcracks at the micro and nano levels, also increases the load transfer within the matrix. This micro and nano levels reinforcement results in a 27.27% increase in flexural strength for the specimen M-GC with respect to specimen PC as shown in Fig. 5b. MWCNTs interact with cement hydration products, particularly calcium hydroxide (Ca(OH)_2_), promoting the formation of additional calcium silicate hydrate (C-S–H) gel. This process strengthens the interfacial transition zone (ITZ), rendering the concrete resistant to cracking. The enhanced bonding at the nanoscale contributes to the increase in the flexural strength^43^. It was found that a 15.8% increase in flexural strength with 0.3% MWCNTs but weak interfacial bonding beyond this threshold is observed^44^. Hence, in the present study 0.1% MWCNT proves to be optimum dosage indicating the uniform dispersion of nano material that enhances strength without necessitating higher MWCNT loadings.

### Effect of elevated temperature

**Fig. 6:**
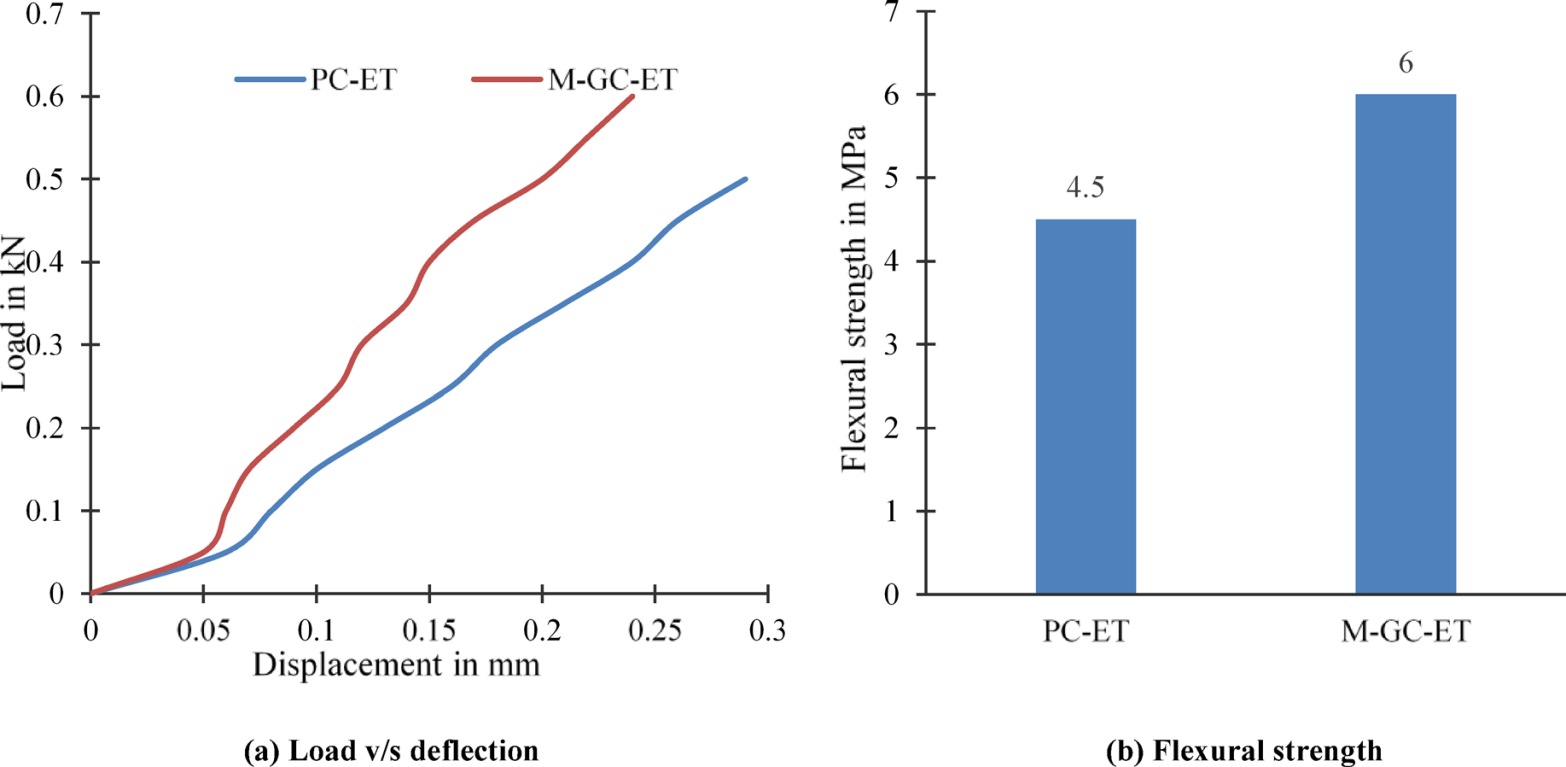
Concrete specimens subjected to elevated temperature.

The elevated temperature induces internal deformation, propagation of microcrack in the concrete structures which reduce the flexural strength of concrete. Figure 6 shows the effect of elevated temperature at 400^0^ C on plain concrete for PC-ET specimen and plain concrete with grouting and coating M-GC-ET. Exposure to elevated temperatures (400 °C) significantly affects the cement matrix by inducing thermal expansion, microcrack formation, and dehydration of calcium silicate hydrate (C–S–H) gel, leading to structural instability and strength reduction. The bond between the cement pastes and aggregate becomes weaker due to the high temperatures, leading to chemical reactions that cause structural deterioration. Figure 6a shows the reduction in the ultimate load of specimen PC-ET compared to specimen M-GC-ET. The integration of MWCNTs and epoxy coatings help mitigate these effects by enhancing thermal resistance. MWCNTs act as nano-reinforcements, effectively bridging microcracks and absorbing tensile stresses at the nanoscale, reducing crack propagation and improving mechanical properties^45^. Additionally, MWCNTs exhibit high thermal conductivity (~ 1600–3200 W/mK), allowing for efficient heat dissipation, which prevents localized thermal stresses that could lead to material degradation. The MWCNT is responsible for the formation of additional calcium silicate hydrate (C–S–H) gel in grouting materials by reacting with calcium hydroxide (Ca(OH)_2_) generated during cement hydration^46^. Therefore, it is found that increment in the flexural strength about 33.33% in the M-GC-ET specimen compared PC-ET specimen when subjected to elevated temperature as shown in Fig. 6b. Also, Epoxy coatings also provide surface-level reinforcement, protecting the underlying material from direct thermal exposure and improving overall mechanical properties for M-GC-ET specimen is compared to PC-ET specimen.

### Influence of concentration of sulfate ions on durability properties

**Fig. 7:**
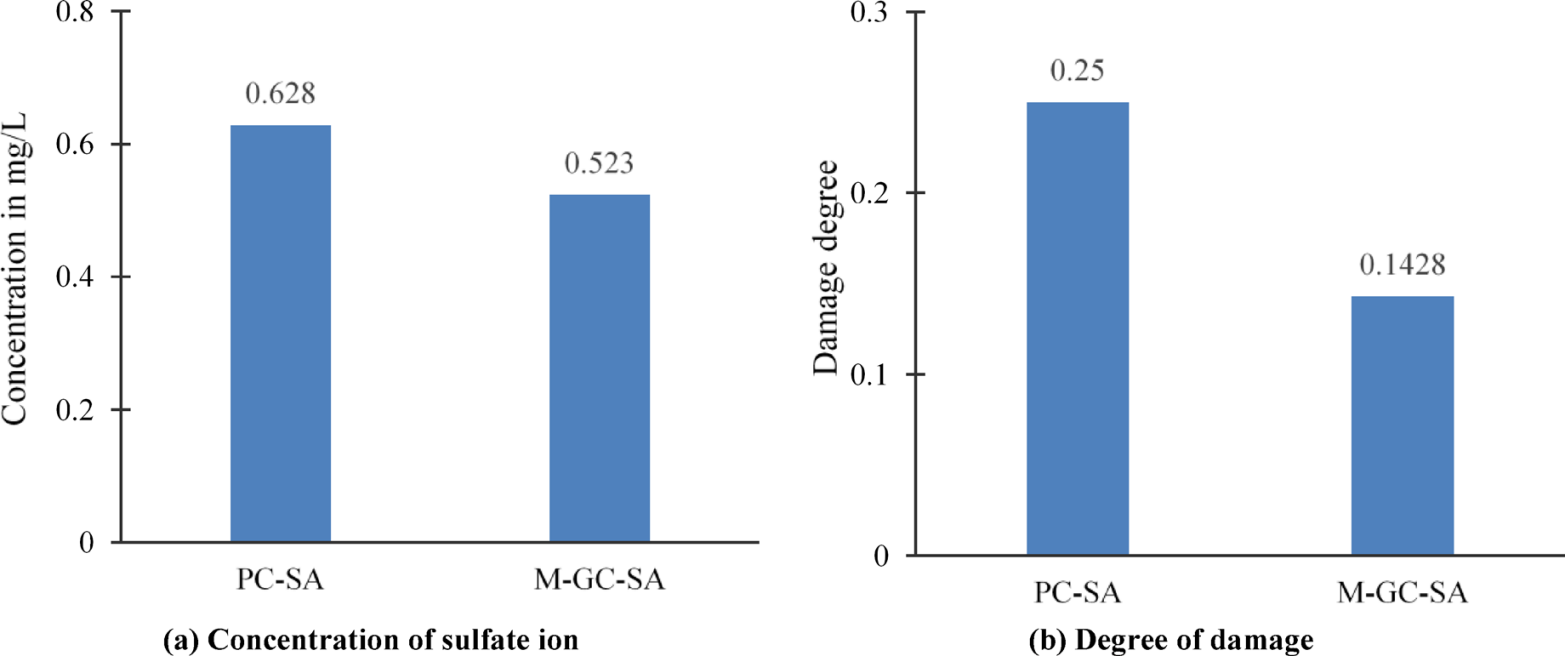
Compressive strength of specimen exposed to chloride attack.

The durability study of a typical concrete structure was conducted based on the critical value of the sulfate ion concentration in the concrete. Concrete’s sulfate ion distribution curve was measured, and the resulting data are shown in Fig. 7. It depicts concentration of sulfate solutions is decaying the concrete durability. Equation (4) is used to analyze the damage mechanism of concrete specimen under sulfate solution environment.

4 $${\text{Damage}}\,{\text{degree}},Di = 1 - \frac{\sigma i}{{\sigma o}}$$

where *Di* is the damage degree of concrete after certain immersing time; σ_*i*_ is the flexural strength of concrete after certain immersing time; σ_0_ is the initial flexural strength of concrete^46^.

Sulfate exposure in concrete structures leads to the formation of products such as gypsum and ettringite, which cause expansion, and cracking, ultimately compromising structural integrity. Experimental study indicates that specimen M-GC-SA retrofitted with MWCNT-based grouting fused with epoxy coatings exhibited higher durability compared to untreated sample PC-SA, which showed significant deterioration. This improvement is attributed to the pore structure modification and chemical interactions of MWCNTs, combined with the protective barrier effect of epoxy coatings. MWCNTs enhance the compactness of the cementitious matrix by filling microcracks and refining the pore structure, thereby reducing sulfate ion ingress and limiting the pathways for aggressive ions, which enhances durability under sulfate.

### Stress v/s Strain characteristics

**Fig. 8:**
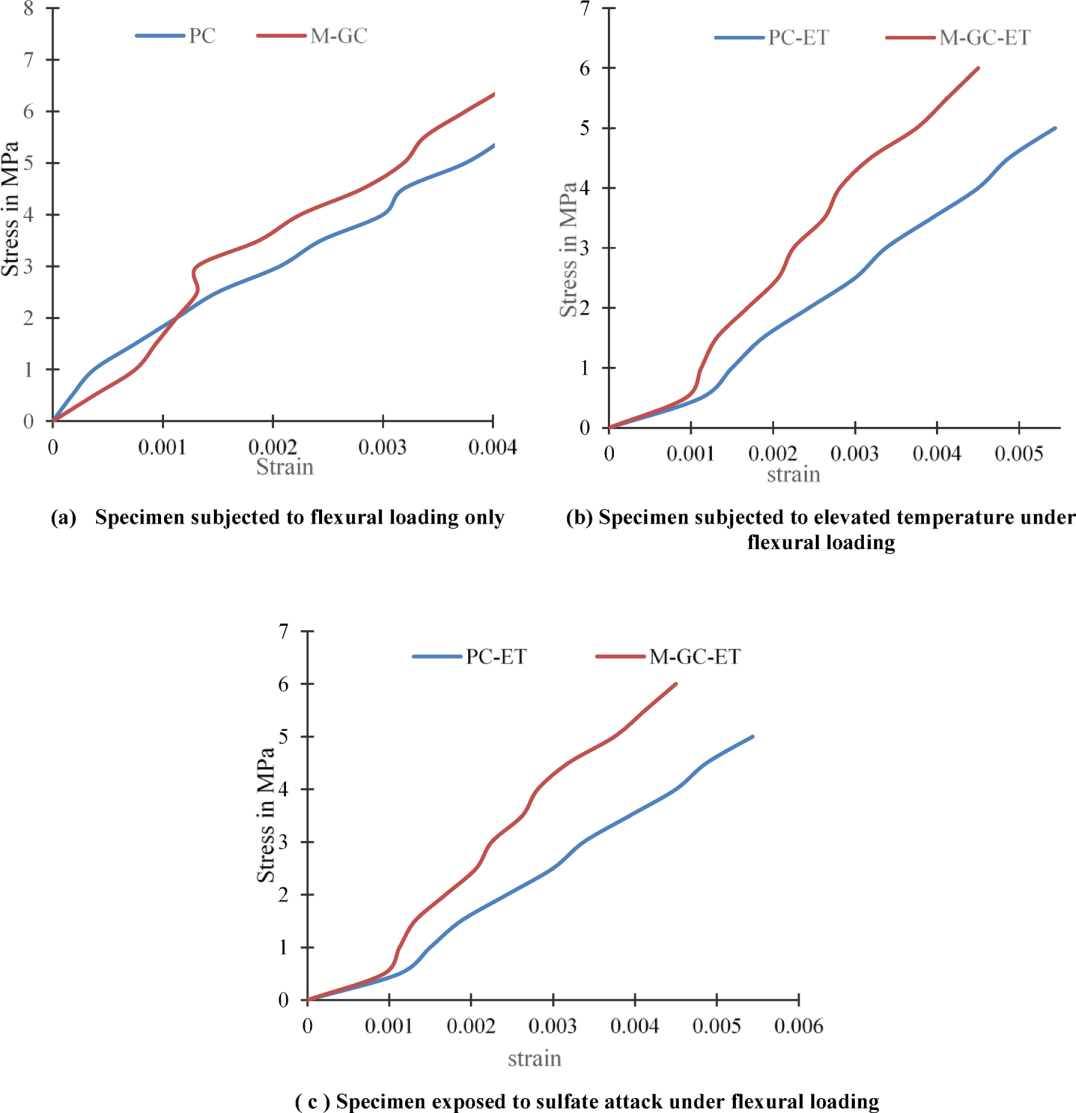
Stress v/s strain diagrams.

The application cement based MWCNT grouting fused with epoxy coating improves the flexural stress–strain relationship for concrete as shown in Fig. 8. MWCNT nano materials improved concrete’s mechanical characteristics by bridging the cracks at both micro and nano levels, preventing their development and better dispersion of loads throughout the material^47^. Their high aspect ratio and mechanical properties reinforce the cementitious matrix, improved structural integrity and performance.

### Flexural toughness

**Fig. 9:**
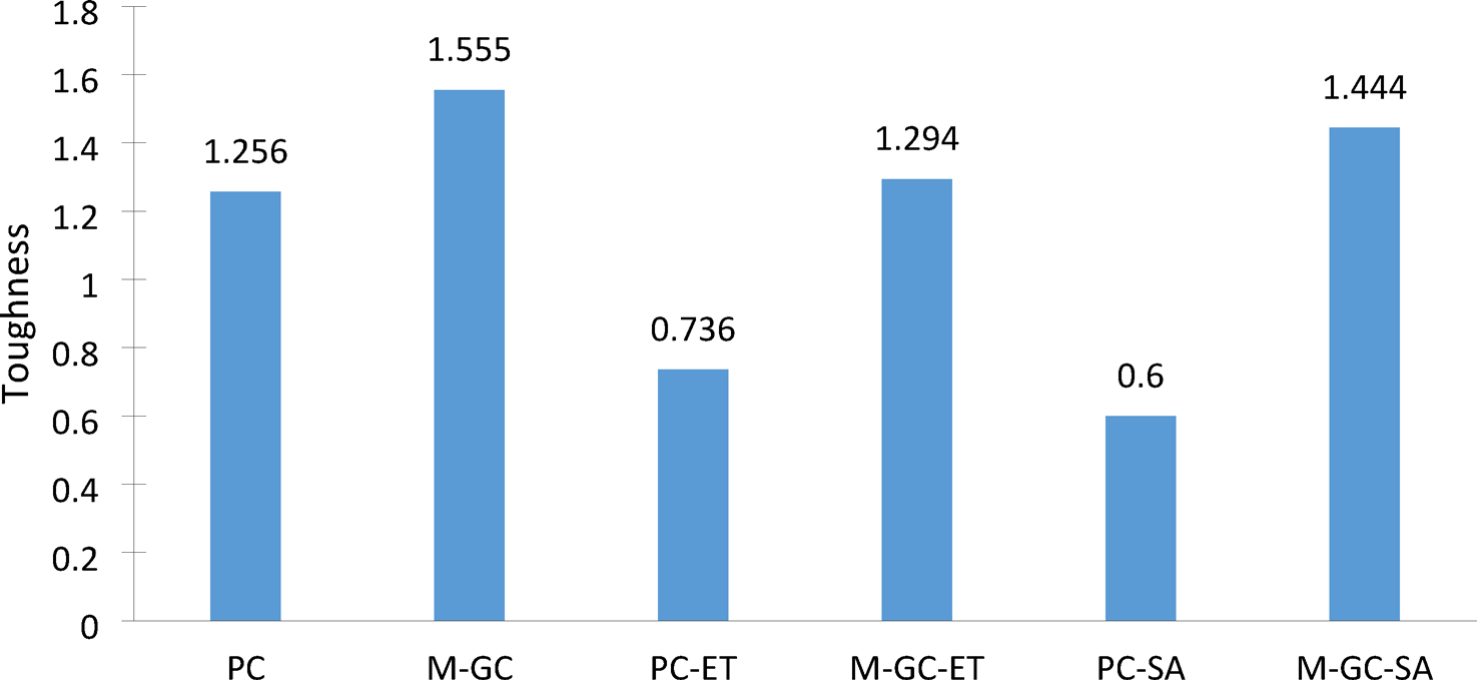
Flexural toughness.

The flexural toughness values of concrete beam specimens at 28 days were calculated by taking area under stress–strain curve^48^. As shown in Fig. 9, when the cracked beam specimens treated with MWCNTs grouting with coating, toughness values were increased. Flexural toughness for specimens named PC-ET, PC-SA, M-GC-ET and M-GC-SA were decreased compared to corresponding values for normal and retrofitted beams. This is because of the elevated temperature and sulfate attack. Elevated temperature reduced concrete’s toughness by thermal expansion, weakening the cement composition and promoting microcracks. This compromises its ability to withstand mechanical stress, leading to decrement in durability and structural integrity. MWCNTs act as reinforcement fibers that absorb energy during deformation, distributing stress more effectively throughout the cementitious matrix. The increased energy dissipation enables the composite to resist sudden failure, which is particularly beneficial in seismic-resistant structures and high-impact applications^49^. In addition, MWCNTs serve as nano-scale crack arrestors, effectively bridging microcracks and impeding their propagation into larger fractures. Their high aspect ratio and exceptional flexural strength facilitate efficient load transfer within the cement matrix, which enhances both toughness of the composite material. Studies have shown that cementitious composites with 0.1–0.3% MWCNTs exhibit up to a 40% increase in fracture toughness due to the synergistic effect of nano reinforcement and enhanced load transfer. In the present study, 0.1% MWCNT shows an increase in the toughness value of about 73% for PC-ET compared to M-GC-ET specimen and 140% M-GC-SA compared to PC-SA specimen respectively.

Sulfate attack on concrete weakens the cement matrix by chemical reactions, causing expansion and cracking. This compromises toughness as the structure becomes more susceptible to deterioration and loss of load-bearing capacity. Moreover, 28 days’ toughness of beam specimens were increased compared to that of the normal mixture. It can be observed in Fig. 9 that, PC-SA specimen achieved the lowest toughness value and highest toughness value was obtained for M-GC specimen.

### Ductility index

According to the energy method, ductility is the capacity to undergo materials to undergo the plastic deformation prior to the failure. It is the ratio of the total energy and the elastic energy. Naaman and Jeong described the Eq. (5) to calculate the ductility index^50^.

5 $$\mu E = \frac{1}{2}\left( {\frac{Et}{{Ee}} + 1} \right)$$

**Fig. 10:**
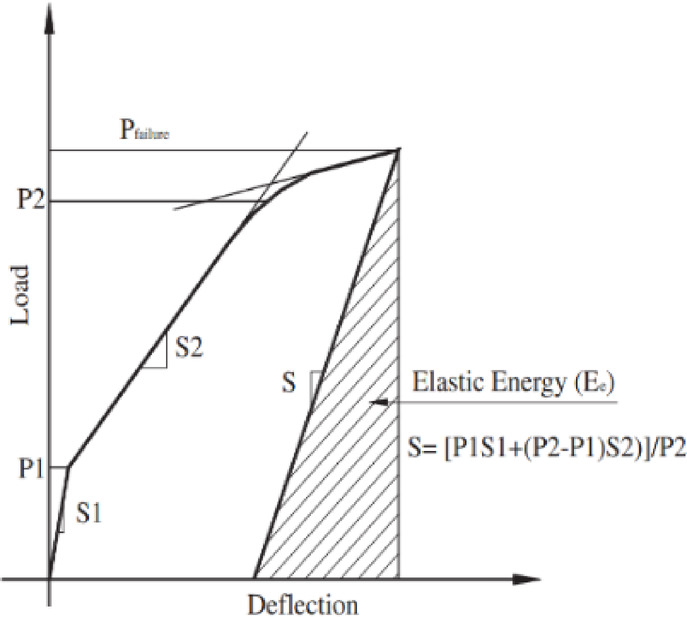
Ductility curve.

**Fig. 11:**
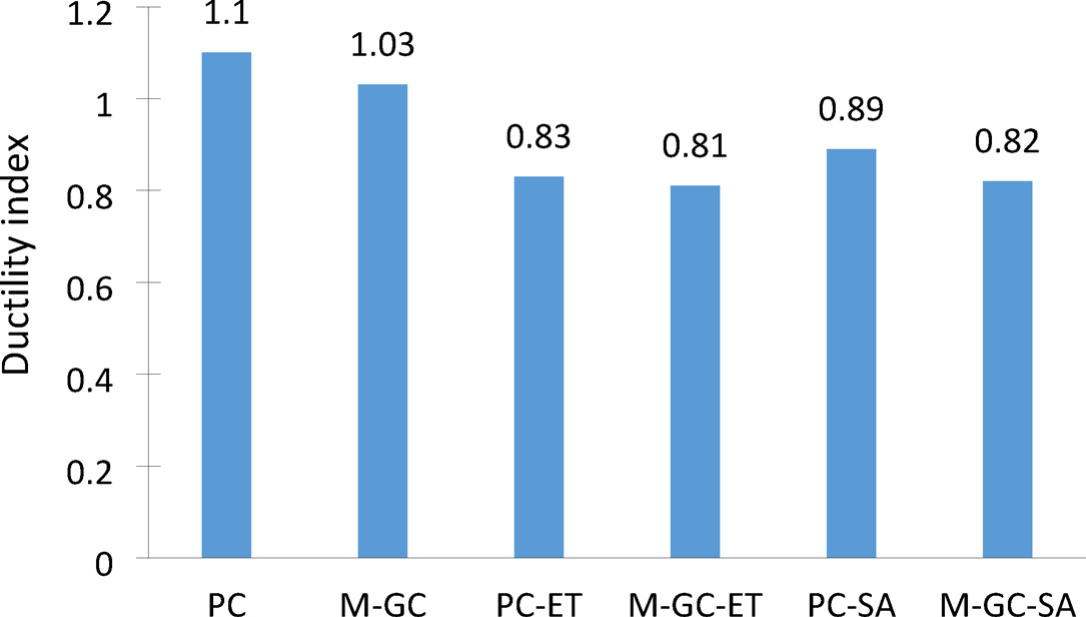
Ductility index.

Here, Et​ represents the total energy, calculated as the area under the load–deflection curve, while Ee​ denotes the elastic energy, determined as the area within line S up to its intersection with P of failure, as illustrated in Fig. 10. Figure 11 shows, decrease in ductility value for a cracked beam specimens treated with MWCNTs grouting with coating. Moreover, as the application of grouting and coating, the ductility values for beam specimens decreased. The addition of MWCNTs chemically interact with calcium hydroxide (Ca(OH)_2_), promoting the formation of additional calcium silicate hydrate (C-S–H) gel, which improves interfacial bonding and consumes Ca(OH)_2_, thereby reducing ettringite formation and mitigating sulfate-induced expansion and cracking leading reduction in ductility^51,52^. In freeze–thaw environments, MWCNTs reduce microcrack formation caused by freeze-induced expansion, improving strength and toughness, while epoxy coatings minimize moisture ingress, preventing internal ice formation^53^. However, epoxy coatings can be susceptible to UV degradation, leading to surface cracking over time, and incorporating UV-resistant additives into the epoxy formulation can improve longevity.

### Modulus of rupture and bending stiffness

**Fig. 12:**
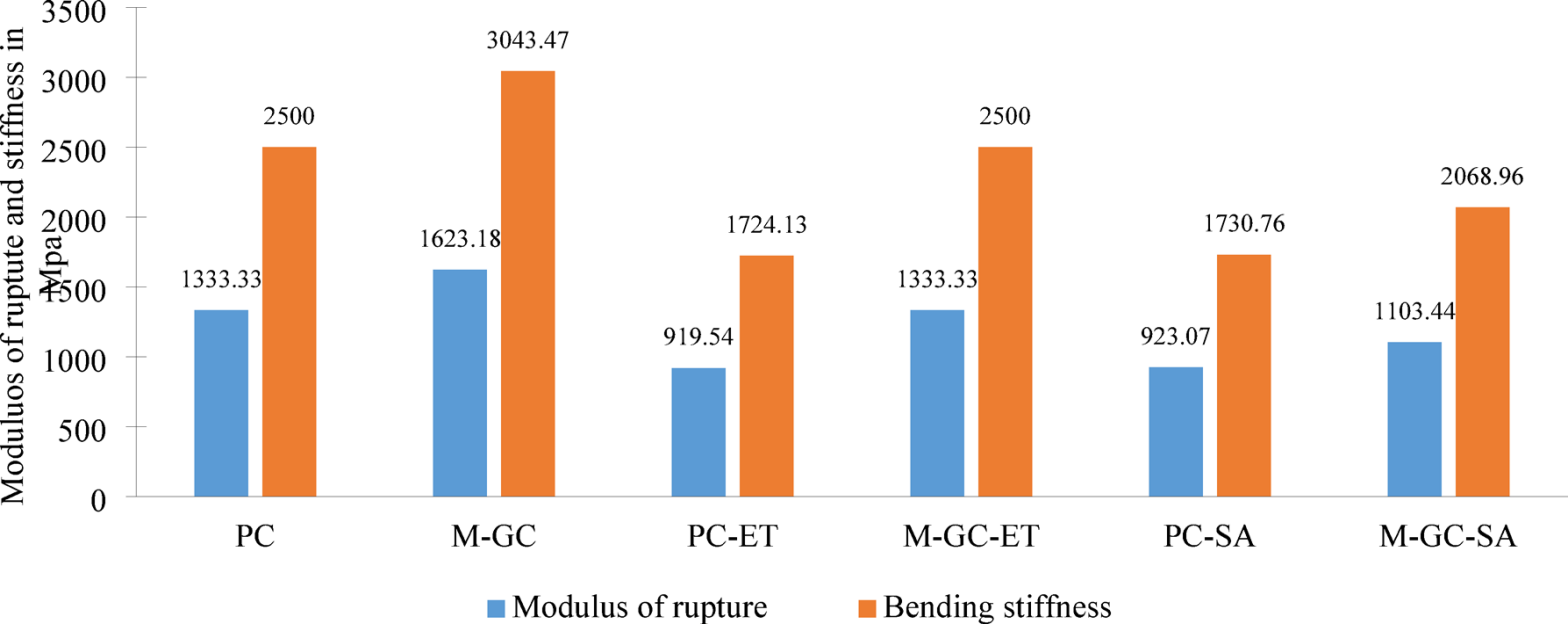
Modulus of rupture and bending stiffness.

Modulus of elasticity and bending stiffness respectively for specimen M-GC, M-GC-ET and M-GC-SA were increased with respect to PC, PC-ET and PC-SA by applying MWCNT grouting and coating as shown in Fig. 12. In concrete beams, the incorporation of MWCNT enhances modulus of elasticity and bending stiffness by reinforcing the matrix at a microstructural level^54^. MWCNT’s high aspect ratio and exceptional mechanical properties disperse loads more effectively, resisting bending deformation and improving the beam’s overall stiffness, thereby enhancing structural performance and durability . It is noted that, MWCNTs mitigate internal cracking by reinforcing nanoscale that increases the modulus of rupture and stiffness. The nano-network formation of MWCNTs enhances load transfer and prevents excessive shrinkage^55^. Furthermore, epoxy coatings act as a moisture retention layer, reducing premature dehydration of the cement matrix which enhance the mechanical strength.

**Fig. 13:**
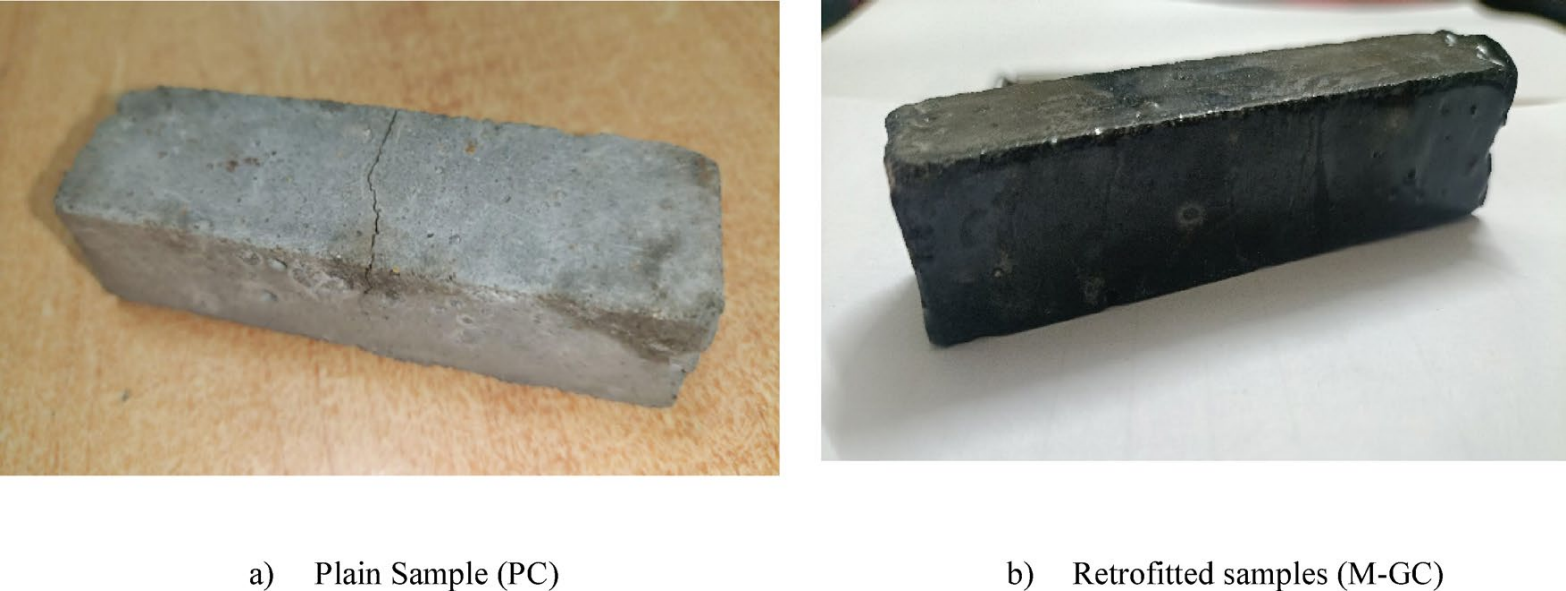
Crack pattern of sample.

Figure 13, depicts the crack and failure patterns for plain concrete (PC) and MWCNT-based grouted & epoxy-coated specimens (M-GC) under flexural loading. The PC specimens exhibited a brittle failure, where cracks initiated at the tension zone and propagated rapidly due to the limited crack-bridging capability of conventional cementitious materials. While, M-GC specimens demonstrated ductile failure, with delayed microcrack formation due to the reinforcing effect of MWCNTs. The cementitious groute infused with MWCNTs filled internal voids, effectively redistributing stress and preventing sudden failure. Hence the proposed novel retrofitting approach demonstrates the synergistic effect of MWCNT enhance the strength of grouting and epoxy coatings enhance crack resistance, delaying failure, and improving long-term durability in extreme environmental conditions.

### Scanning electron microscope analysis

**Fig. 14:**
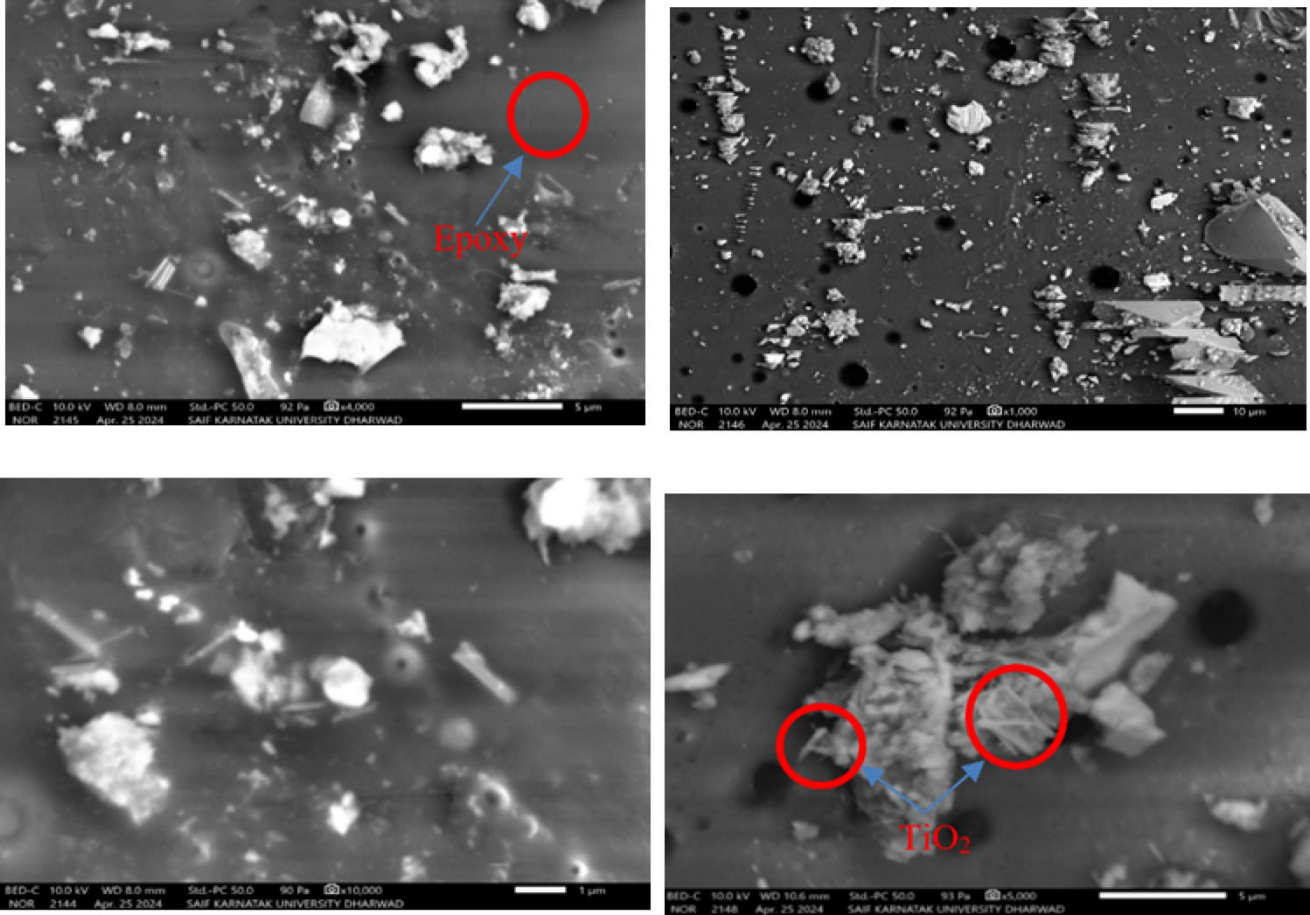
SEM images of concrete specimens.

The SEM analysis provides the initiation, development induced cracks in concrete during the loading phase. The formation of theses cracks reduces the effective load-bearing surface, increasing stresses at crucial break locations. The use MWCNT adds resistance to crack propagation by filling up cracks and pores. MWCNTs have a strong fracture-bridging action under loading, which helps to prevent crack propagation in concrete structures. Additionally, the presence of MWCNTs promoted the formation of secondary calcium silicate hydrate (C–S–H) gel by acting as nucleation sites, which significantly strengthened the interfacial transition zone (ITZ) and increased both compressive and flexural strength^56^. The SEM images further indicated that the well-dispersed and aligned MWCNTs formed a reinforcing network within the cement matrix, improving load transfer efficiency and mitigating microcrack propagation. Proper dispersion and alignment of MWCNTs played a crucial role in preventing agglomeration, ensuring uniform stress distribution, and enhancing toughness^57^. The double sonication method employed in this study facilitated better uniformity, optimizing the reinforcing effect of MWCNTs. Furthermore, the chemical interaction between MWCNTs and hydration products, particularly calcium hydroxide (Ca(OH)_2_), contributed to the densification of C–S–H gel, leading to enhanced interfacial bonding strength and long-term durability of the cementitious composite. The epoxy resin hinders the diffusion of grouting materials and seals pores present in cement matrix. Therefore, it enhances strengthening and crack-bridging of grouting material as shown in Fig. 14. Hence, SEM analysis illustrates the potential of nano reinforcements to improve the mechanical characteristics and longevity of concrete structures.

## Conclusions

This study investigates the synergistic effects of cementitious nanocomposites grouts fused with epoxy coatings for structural retrofitting applications. This technique is effectively used for cracked concrete structures to enhance the mechanical properties such as load carrying capacity, resistance to deformation and durability for retrofitting of structures. The experimental findings demonstrated an improvement in rupture strength of about 27.27% after restoring cracks in plain concrete using MWCNT with grouting fused with epoxy coating. Similarly, the improvement in rupture strength found to be 33.33 and 20% for concrete specimen after restoring cracks the subjected elevated temperature of 400 °C and sulfate attack respectively. The nano-reinforcement effect of MWCNTs contributed to enhanced crack bridging, stress redistribution, and load transfer, effectively improving modulus of rupture and bending stiffness. The failure pattern analysis shows the. transition from brittle failure in plain concrete specimens to ductile failure in M-GC specimens, indicating improved toughness and energy absorption ability due to MWCNT reinforcement. Thermal and durability assessments revealed significant improvements in high-temperature resistance and sulfate exposure durability for retrofitted samples. The plain concrete specimens show severe crack widening while M-GC specimens show reduced crack widths, confirming the thermal stability imparted by MWCNTs and the protective role of epoxy coatings. Similarly, sulfate-exposed PC-SA specimens exhibited visible surface deterioration, whereas M-GC-SA specimens retained their integrity, demonstrating the barrier effect of epoxy coatings in preventing sulfate ion ingress. Application of epoxy coating after the grouting on concrete surface provides an additional layer of protection and resistance to sulfate ion penetration. Hence, epoxy coatings offer a resilient, impermeable barrier that protects concrete structures from external environmental factors and chemical attacks. The microstructural analysis (SEM) confirmed that MWCNTs improved interfacial bonding, refined C–S–H gel formation, and acted as nano-reinforcements, leading to improved mechanical properties and crack resistance. The failure pattern analysis revealed that the MWCNT-reinforced grout delayed crack propagation and redistributed stress, ensuring enhanced post-crack behavior. Thus, MWCNT grouting and epoxy coating combined can greatly improve the concrete structure’s durability and structural performance, resulting in an effective restoring of cracks. The proposed techniques possess a real-world application in adverse environmental conditions, including marine structures, wastewater treatment plants, and sulfate-rich soil environments. Future research should focus on evaluating the long-term durability of MWCNT-based grouting fused with epoxy coatings under diverse environmental and loading conditions such as cyclic loading, freeze–thaw cycles, carbonation, and chloride ingress in marine environments. In addition, field-scale investigations are essential to validate the laboratory findings and establish practical guidelines for structural engineers. The integration of smart sensing systems within the grouting coating framework could provide real-time monitoring of crack propagation and durability performance, further enhancing its applicability. Moreover, assessing the sustainability, cost-effectiveness, and life-cycle benefits of this hybrid retrofitting strategy compared to conventional methods would strengthen its potential for large-scale adoption in infrastructure rehabilitation projects.

## Data Availability

The data presented in this study can be made available on request from the corresponding author.
